# A Single Education Session of Orthopaedic Residents Does Not Reduce The Rate of Failed Nonoperative Management or Improve Radiographic Outcomes in Pediatric Distal Radius Fractures

**DOI:** 10.5435/JAAOSGlobal-D-20-00170

**Published:** 2020-10-16

**Authors:** Edward Compton, Adrian Lin, Kenneth D. Illingworth, Melissa A. Bent

**Affiliations:** From the Children's Orthopaedic Center, Children's Hospital Los Angeles, Los Angeles, CA.

## Abstract

**Introduction:**

The primary objective was to evaluate whether a single educational session on casting is sufficient to reduce the rate of loss of reduction in pediatric distal radius fractures.

**Methods:**

A retrospective review was conducted of pediatric patients with distal radius fractures casted between November 2016 and February 2019. Patients were divided into two groups: those casted by a resident who participated in a targeted education session on short arm casting and those who had not.

**Results:**

A total of 137 patients were included (education cohort: 61 patients and noneducation cohort: 76 patients). The two groups demonstrated similar ages and pre/post-reduction radiographic measurements. In the education cohort, 11.5% required repeat casting, wedging, or surgical intervention versus 17.1% of patients in the noneducation cohort (*P* = 0.47). Patients casted by residents doing one of their first three independent casts trended toward being more likely to place a cast with poor cast index and to lose reduction (*P* = 0.12 and *P* = 0.43, respectively).

**Discussion:**

A one hour education session did not reduce the need for intervention or loss of reduction. For educating residents on the skill of casting to be effective, one may consider formal feedback and evaluation throughout multiple education sessions and in early episodes of clinical care.

**Level of Evidence:**

A Level III, Retrospective Comparative Study

Distal forearm fractures account for up to 36% of all pediatric fractures, with a peak incidence in male patients 12 to 14 years old and female patients 10 to 12 years old.^1,2,3,4,5,6^ The standard of care for most distal radius fractures is closed reduction and cast immobilization because up to 85% of patients with displaced fractures will heal appropriately without the need for surgical intervention.^7,8^ Approximately one-third of distal radius fractures can have a loss of reduction or redisplacement.^9,10^ Risk factors for redisplacement include failure to achieve an anatomical reduction, isolated radius fractures without a concomitant ulnar fracture, complete displacement of the fracture in any plane, bivalving a cast acutely after placement, and cast quality, which includes cast index, three-point index, and appropriate ulnar deviation.^9,11,12,13,14,15^ Cast quality is a modifiable risk factor and can have a notable impact on success of nonoperative management.

The Accreditation Council for Graduate Medical Education (ACGME) has identified principles and techniques of fracture reduction, casting, and splinting as a priority in the surgical skills curriculum for orthopaedic surgery interns. Several efforts exist to incorporate simulation modules into residency curriculums, with the potential benefit of allowing residents to learn and practice skills in a low-risk environment without potential patient harm, time constraints, and the ability to repeat the skill while receiving feedback from more senior colleagues. Previous studies have demonstrated that a decrease exists in subsequent loss of reduction and improved cast molding measured by cast index and three-point index with use of simulation models.^16,17,18,19,20,21^ Procedure-based curricular development, especially with the rapid incorporation of education sessions and simulation training into residency programs, lack uniformity across ACGME programs. A previous study that examined outcomes of pediatric distal radius fractures found that loss of reduction was common, especially when junior residents were doing their first few independent reductions. However, residents who underwent a simulation training module had a lower rate of subsequent loss of reduction, highlighting the value of a simulation module as a supplement to traditional training methods.^22^

Our primary aim was to determine whether the rate of loss of reduction in pediatric distal forearm fractures is affected by a single targeted cast education session consisting of a didactic lecture and subsequent practical cast application session without a simulation model compared with no education and practical cast application session. The secondary objective was to characterize residents' self-perception of their casting skills.

## Methods

### Patient Data

After institutional review board approval was granted for this study, a retrospective review of patients aged 1 to 17 years old with distal radial fractures treated nonoperatively with closed reduction and casting from November 2016 to February 2019 at a tertiary pediatric hospital was conducted. Exclusion criteria included patients initially treated with splinting, surgical intervention, those without adequate pre- and post-reduction radiographs, and patients without at least one follow-up radiograph.

Patients were divided into two groups: those casted by a resident who had undergone a single 60 minute educational session (education cohort) consisting of a didactic lecture and practical group casting session (January 2018 to February 2019) and those who had not (noneducation cohort) (November 2016 to January 2018). Since implementation of the education session in January 2018, all residents who rotated at our institution were required to participate in the educational session. Before this, the educational session was not offered. Residents participated in this educational session at the beginning of their rotation each time they rotated at our institution, with rotations lasting from 4 to 12 weeks. Electronic medical records were reviewed for patient age, level of training of the resident who did the reduction and casting, fracture reduction and cast immobilization date, and cast complications including loss of reduction requiring repeat reduction and recasting, cast wedging, or surgical intervention. Junior residents were defined as postgraduate year (PGY)-2 and PGY-3. Senior residents were defined as PGY-4 and PGY-5. Pre- and post-reduction radiographs were assessed for fracture pattern, AP and lateral angulation and displacement, and postreduction cast index. Cast index was defined as the cast width on a lateral radiograph divided by the cast width on an AP radiograph. As previous studies have shown that a cast index ≥0.81 is a risk factor for redisplacement, an acceptable cast index was defined as <0.81.^12,13,23^ Residual angulation and displacement measurements were defined as postreduction angulation/displacement divided by prereduction angulation/displacement. Patients without a prereduction deformity were excluded from residual measurements. Radiographs at the first follow-up appointment were assessed for loss of reduction. Loss of reduction for patients <9 years old was defined as >20° sagittal angulation or >10° of coronal angulation. In patients ≥9 years old, loss of reduction was defined as >10° of sagittal angulation or >10° of coronal angulation.^24^ Clinically notable loss of reduction was defined as loss of reduction, which required repeat reduction and casting, cast wedging, or surgical intervention.

Using the same series of reductions and castings as above, a separate analysis was conducted to evaluate the serial performance of individual residents. Residents who had did at least five reductions and castings in this series were identified. Residents were excluded from this analysis if they attended the education session between their first and last closed reduction and casting.

### Casting Curriculum

Residents participated in a short arm cast curriculum consisting of a 12-minute video on casting application and removal before the session, a single 30-minute didactic lecture, and a 30-minute practical hands on casting session done the following week. No simulation model was used. Junior (PGY 2 and 3) and senior (PGY 4 and 5) residents were paired in groups of two. On average, there were 4 residents per session (2 groups). The sessions were instructed by primarily by a pediatric orthopaedic attending. Residents were assessed by a modified International Pediatric Orthopaedic Symposium top gun simulation scoring sheet (Appendix) that assessed precast plan, proper cast application, and safe cast removal. Total time for the education session was 72 minutes (including the video before the 60-minute session).

### Resident Survey

Orthopaedic surgery residents rotating at our institution from January 2018 to October 2019 were administered an initial survey at the beginning of their pediatric orthopaedic rotation. These residents are from three different residency training programs. Residents were asked their level of training, self-evaluation of their casting ability, previous training in casting technique, and the most important skill they wanted to work on.

### Statistics

A Student *t*-test was used to examine the relationship between casting groups and quantitative variables. A Fisher exact test or a Pearson chi-squared test was used to compare the categorical variables. Residents with at least five reductions before or five reductions after the implementation of the casting curriculum were analyzed to determine whether rates of loss of reduction and unacceptable cast indexes were different between a resident's first three reductions and the resident's subsequent reductions. Risk ratios (RRs) and 95% confidence intervals (CIs) were calculated. Statistical significance was defined as *P* < 0.05. Statistical analysis was done using STATA/1C 14.0 (Stata Statistical Software: Release 14; StataCorp LP, 2015, College Station, TX).

## Results

### Radiographic Measures/Indices

#### AP Displacement/Lateral Angulation

A total of 137 patients with distal radius fractures (mean age: 9.1 ± 3.2 years; range, 1 to 17 years) met the inclusion criteria. Of these, 61 patients were in the education cohort (mean age: 9.6 ± 3.3 years), whereas 76 patients were in the noneducation cohort (mean age: 8.7 ± 3.1 years) (*P* = 0.14). No significant difference was noted in fracture pattern between the education cohort and the noneducation cohort (*P* = 0.07) (Table 1). All patients were casted in fiberglass. Around 67.2% (41/61) of patients in the education cohort had a concomitant distal ulnar fracture, whereas 73.7% (56/76) of patients in the noneducation cohort had a concomitant distal ulnar fracture (*P* = 0.41). One hundred twenty-eight patients had documentation of the treating resident's level of training. Around 17.5% (10/57) of patients in the education cohort were casted by junior residents, whereas 14.1% (10/71) of patients in the noneducation cohort were casted by junior residents (*P* = 0.63) (Table 1). No difference was noted in the length of follow-up between the two groups (*P* = 0.95).

**Table 1:** Patient Characteristics and Fracture Pattern

	Education Cohort (N = 61)	Noneducation Cohort (N = 76)	*P* Value
Age (yrs)	9.6 ± 3.3	8.7 ± 3.1	0.14
Concomitant distal ulnar fracture	41 (67.2)	56 (73.7)	0.41
Mean follow-up time (mo)	2.4 ± 2.3	2.4 ± 3.8	0.95
Type of radial fracture			
Transverse	50 (82.0)	56 (73.7)	0.07
Oblique	6 (9.8)	5 (6.6)	
Salter harris	4 (6.6)	15 (19.7)	
Volar sheer	1 (1.6)	0 (0.0)	
Resident level of training			
Junior	10 (17.5)	10 (14.1)	
Senior	47 (82.5)	61 (85.9)	0.63

Values are presented as mean ± SD or n (%).

No difference was noted in prereduction AP displacement and angulation (*P* = 0.73 and *P* = 0.33, respectively) between the two groups, and no difference was noted in prereduction lateral displacement and angulation (*P* = 0.37 and *P* = 0.83, respectively) (Table 2). No difference was noted in postreduction AP displacement and angulation (*P* = 0.71 and *P* = 0.37, respectively) between the two groups, and no difference was noted in postreduction lateral displacement and angulation (*P* = 0.79 and *P* = 0.84, respectively) (Table 2). No difference was noted between the two groups in residual AP displacement (*P* = 0.48), residual lateral angulation (*P* = 0.78), and residual lateral displacement (*P* = 0.39) (Table 2). A significantly lower residual AP angulation was observed in the education trained cohort (9.8% ± 16.5%) than in the noneducation cohort (22.7% ± 42.3%) (*P* = 0.04).

**Table 2:** Pre-R, Post-R, and Residual AP and Lateral Angulation and Displacement

Physeal/Bicortical	Education Cohort (N = 61)	Noneducation Cohort (N = 76)	*P* Value
Pre-R AP angulation (°)	13.7 ± 10.0	12.0 ± 10.9	0.33
Pre-R AP displacement (mm)	4.6 ± 3.5	4.8 ± 3.8	0.73
Pre-R lateral angulation (°)	18.6 ± 13.6	18.1 ± 14.1	0.83
Pre-R lateral displacement (mm)	7.3 ± 4.4	8.0 ± 3.9	0.37
Post-R AP angulation (°)	1.4 ± 2.6	1.8 ± 3.1	0.37
Post-R AP displacement (mm)	1.4 ± 1.4	1.5 ± 1.4	0.71
Post-R lateral angulation (°)	3.9 ± 4.3	3.7 ± 4.2	0.84
Post-R lateral displacement (mm)	1.4 ± 1.3	1.5 ± 1.8	0.79
Residual AP angulation (%)	9.8 ± 16.5	22.7 ± 42.3	0.04*
Residual AP displacement (%)	37.9 ± 34.6	43.6 ± 46.9	0.48
Residual lateral angulation (%)	51.3 ± 108.7	45.3 ± 101.2	0.78
Residual lateral displacement (%)	27.7 ± 39.1	21.8 ± 30.9	0.39

Post-R = postreduction; Pre-R = prereduction

* Significance at *P* < 0.05.

Values are presented as mean ± SD.

#### Cast Indices

The average cast index in the education cohort was 0.78 ± 0.09, whereas in the noneducation cohort, the average cast index was 0.79 ± 0.08 (*P* = 0.66). No significant difference was noted between groups when comparing cast indices ≥0.81 (education cohort: 39.3%, 24/61 versus noneducation cohort: 46.1%, 35/76; *P* = 0.43). No significant difference was noted between junior and senior residents when comparing cast indices ≥0.81 (junior: 40.0%, 8/20 versus senior: 44.4%, 48/108; *P* = 0.71). Patients casted by junior residents (35.0%; 7/20) lost reduction at similar rates as patients casted by senior residents (23.1%; 25/108) (*P* = 0.27).

#### Follow-up

The average time to the first follow-up visit was similar between the education cohort (10.8 ± 7.8 days) and the noneducation cohort (10.9 ± 8.0 days) (*P* = 0.90). Around 24.6% (15/61) of patients in the education cohort lost reduction, whereas 27.6% (21/76) of patients in the noneducation cohort lost reduction (*P* = 0.69). Around 11.5% (7/61) of patients in the education cohort lost reduction and subsequently underwent repeat reduction and casting (N = 4) or surgical intervention (N = 3), whereas 17.1% (13/76) of patients in the noneducation cohort lost reduction and subsequently underwent repeat reduction and casting (N = 7), cast wedging (N = 5), or surgical intervention (N = 1) (*P* = 0.47). Surgical interventions are summarized in Table 3. One patient requiring surgical intervention was originally casted by a junior resident, whereas the other three were originally casted by a senior resident. All patients requiring surgical intervention had isolated distal forearm fractures without additional injuries.

**Table 3:** Surgical Intervention for Fracture Loss of Reduction

Surgical Intervention	Education Cohort (Y/N)	PGY Level of Resident Doing Initial Reduction	Fracture Type	Cast Index	Age (yrs)
ORIF	N	4	Oblique	0.73	10
ORIF	Y	2	Volar sheer	0.79	15
CRPP	Y	4	Transverse	0.84	11
CRPP	Y	4	Transverse	0.72	10

CRPP = closed reduction and percutaneous pinning; ORIF = open reduction and internal fixation; PGY = postgraduate year

#### Serial Performance of Individual Residents

Among these 137 closed reduction and castings, five residents were identified who had did ≥5 closed reductions and cast immobilizations during patient encounters. Three of these residents did their reductions before the implementation of the education session, and two residents did their reductions after the implementation of the education session. Forty percent (6/15) of patients in a resident's first three closed reductions and cast immobilizations lost reduction, whereas 30% (7/23) of subsequent patients lost reduction. Patients were 1.3 times as likely to experience a loss of reduction in a resident's first three closed reductions and cast immobilizations than in their subsequent closed reductions and castings (RR, 1.3; 95% CI, 0.58–2.8; *P* = 0.43). Seventy-three percent (11/15) of patients in a resident's first three closed reductions and cast immobilizations had casts placed with a cast index ≥0.81, whereas 48% (11/23) of subsequent patients had casts placed with a cast index ≥0.81. Patients were 1.5 times as likely to have a cast placed with a cast index ≥0.81 in a resident's first three closed reductions and castings than in their subsequent closed reductions and castings (RR, 1.5; 95% CI, 0.91–2.6; *P* = 0.12). In the four patients who required surgical intervention, 75% were during a resident's first threes casts on the rotation. The fourth resident, although it had been the sixth cast on rotation, half of that resident's casts resulted in loss of reduction.

#### Resident Survey

Thirty-eight residents completed the survey between February 2018 and October 2019 (Table 4). Residents provided feedback into what they viewed as the most useful casting skills. Around 52.6% (20/38) cited “basics” and “reduction techniques” as skills they would like to improve. Around 42.1% (16/38) of residents thought that patient case review would be helpful. Senior residents (90.1%) were more likely to report experience with fiberglass casting than junior residents (73.1%), although this was not significant (*P* = 0.63). Overall, 21.1% of respondents reported no previous experience with fiberglass casting. Regarding self-rating of casting technique, 55.3% (21/38) considered their cast technique “proficient,” 23.7% (9/38) considered their cast technique “needs improvement,” and 2.6% (1/38) considered their technique “poor.” No significant difference was noted between level of training and how residents reported their casting technique (*P* = 0.13; Table 4), although no senior residents felt that their casting technique needed improvement. Around 65.8% (25/38) of residents had received formal training mainly during intern year in casting technique before rotating at our institution, whereas 34.2% (13/38) had not received previous formal training. No difference was noted between level of training and having received previous formal casting technique training (*P* = 0.29). Eighty-seven percent (33/38) of residents thought that a formal comprehensive pediatric casting curriculum would be useful, whereas 13.2% (5/38) thought that a formal curriculum would not be useful without significant difference (*P* = 0.21).

**Table 4:** Results of Resident Survey Conducted on Perception of Casting Ability and Necessity for Casting Curriculum

Question	Answer	PGY-2 (N = 7)	PGY-3 (N = 19)	PGY-4 (N = 8)	PGY-5 (N = 4)	*P* Value
How do you consider your cast skills?	Superior	0 (0)	3 (15.8)	2 (25.0)	2 (50.0)	
	Proficient	3 (42.8)	10 (52.6)	6 (75.0)	2 (50.0)	
	Needs improvement	3 (42.8)	6 (31.6)	0 (0)	0 (0)	
	Poor	1 (14.2)	0 (0)	0 (0)	0 (0)	0.13
Have you had formal training in casting?	Yes	3 (42.9)	15 (78.9)	5 (62.5)	2 (50.0)	
	No	4 (57.1)	4 (21.1)	3 (37.5)	2 (50.0)	0.29
Do you think a formal comprehensive pediatric casting curriculum would be helpful?	Yes	7 (100)	14 (73.7)	8 (100)	4 (100)	
	No	0 (0)	5 (26.3)	0 (0)	0 (0)	0.21
Previous experience with casting material	Plaster	1 (14.3)	5 (26.3)	1 (25.0)	0 (0)	
	Fiberglass	5 (71.4)	14 (73.7)	6 (75.0)	4 (100)	
	Orthoglass	1 (14.3)	0 (0)	0 (0)	0 (0)	0.63

PGY = postgraduate year

Values are n (%).

## Discussion

The present study is an evaluation of a single 60-minute resident education casting session on patient outcomes in pediatric distal forearm fractures. We found that residents who participated did not have improved postreduction angulation, translation, or cast indices when compared with residents who had no formal educational session at our institution. Only residual AP angulation showed improvement between those who had attended the education session and those who had not. This indicates that the single education session was not sufficient to notably impact patient outcomes for distal radius fractures. Both groups had mean acceptable cast indexes <0.81 independent of level of seniority.^12,13^ The rate of loss of reduction was similar between the pre-education-trained group and in the education-trained group, independent of the level of training. Patients who lost reduction who underwent remanipulation or surgical intervention were equal between the two groups, indicating that the single education session did not improve outcomes of patients with distal radial fractures. In three of the four patients who required surgical intervention, the cast was one of the first three casts placed by the resident on their rotation at our institution. This highlights that a learning curve likely exists throughout the rotation and that the first reduction by a resident may be a risk factor for failure of nonoperative treatment.

Our study demonstrated that junior residents did similar to senior residents. The more important factor was related to clinical experience of casting during the rotation. Patients casted by residents doing one of their first three independent casts had a trend toward being more likely to a cast placed with a poor cast index and to lose reduction. Patients were 1.3 times as likely to have a loss of reduction and 1.5 times as likely to have a cast placed with an unacceptable cast index in a resident's first three reductions than in their subsequent reductions. Although this did not reach significance, this suggests that at the beginning of a resident's rotation, more diligent supervision may be necessary. Our findings are similar with a prospective analysis of 103 patients with distal forearm fractures that were randomized into groups to be treated by an orthopaedic surgery resident or a pediatric emergency medicine fellow/attending who participated in a didactic lecture and subsequently, had their first five reductions under the supervision of a pediatric orthopaedic surgeon. No notable differences existed between those reduced and casted by pediatric emergency physicians and orthopaedic residents, indicating that goal-directed educational sessions, coupled by close supervision by a qualified superior until a novice trainee (regardless of training specialty) should be incorporated into the development of educational curricula.

Competing interests exist for resident's time. Reduction in resident working hours resulting in fewer patient encounters, increased surgical management of distal radius fractures, and increased use of orthopaedic physician extenders for casting has reduced the opportunity for resident casting.^25-27^ Simulation modules have been developed that aim to increase resident exposure and allow for objective feedback and repetition. The impact of simulation modules on patient outcomes have been examined by Bae et al and Jackson et al.^16,22^ Both studies had a lecture on cast technique and used a simulation module incorporated into their curriculum with 2.5 to 3 hours of additional training with feedback by senior orthopaedic faculty. Both investigated the impact on different patient outcomes. Bae et al saw a notable reduction in the rate of cast-saw burn injuries from 4.3% before simulation to 0.7% after simulation training was implemented. Similarly, Jackson et al found that residents that underwent simulation training had lower residual AP angulation, lateral translation, and lower rates of loss of reduction. They also demonstrated that with more reductions and casting on real patients, residents were less likely to lose reduction. The curriculum proposed by Bae et al and Jackson et al seems to be advantageous in that they offer residents an opportunity to practice reductions in a well-controlled environment on a simulation model with adequate time and allowed for feedback.

Three distinct features exist that differentiate our education session and those previously described: (1) the duration of the education session, (2) the use of a simulation model, and (3) meaningful clinical feedback and supervision. In the previous studies, the duration of the education sessions lasted at least 2.5 hours, whereas in our education session, the duration was only 30 minutes for hands on cast application. This implies that the time allotted to our residents to learn and master their casting technique was not sufficient. In the previous studies, residents repeated casting on simulation models and used imaging to provide real-time feedback from senior colleagues or attending orthopaedic surgeons. This underscores the necessity to repeat a learned skill to fully master a technique. Consistent clinical feedback after the session in patient care perhaps is as important as the actual education session to further expand on fundamentals. As residency program directors plan education sessions for their residents, it is unlikely that a 60-minute education session without clinical feedback is sufficient to improve patient outcomes. It is therefore imperative that program directors allocate adequate time and resources for residents to develop their skills.

Our study also suggests a lack of standardization in the training casting skills to orthopaedic surgery residents. Only 66% of residents had received previous formal training in casting despite this being one of the ACGME's required skills in the intern curriculum. In addition, 21% of residents lacked any experience with fiberglass casting. Because most casts were placed by senior residents and residents who already had formal training in cast application, the residents were likely well versed in the parameters of a well-molded cast. However, 87% of surveyed residents, including 100% of senior residents, felt that a formal pediatric casting curriculum would be useful, highlighting the residents' lack of comfort in dping these reductions and castings. These findings highlight that seniority alone does not portend comfort and ease of reductions and casting in pediatric patients.

The lack of standardization in resident curriculum may contribute to the variability in resident performance in basic orthopaedic techniques. Various training programs approach casting education differently. Some programs have their residents shadow cast technicians, whereas others are led by a senior resident or attending. Thus, benchmarks need to be met demonstrating competency in casting before allowing residents to do them regularly. Implementation of a formal standardized curriculum could help to alleviate some of the discrepancies in reduction and casting technique because most residents surveyed felt that this would be useful.

Kern et al described a six-step model for a systematic method for curricular development.^28^ Khamis et al^29^ adapted this model further, developing a systematic guideline for curricular development regarding an effective simulation design by evaluating features of successful simulation modules. The adoption of best-practice guidelines for curricular development becomes increasingly paramount to ensure adequate and equitable training across all trainees as focused educational sessions and simulation-based educational modules are becoming increasingly prevalent.

Several limitations exist to this study. First, it is limited by its retrospective nature. Incomplete documentation or lack of routine follow-up may limit the scope of our findings. Second, almost two-thirds of the residents surveyed had undergone a formal casting training before rotating at our institution. These were the same residents who participated in the formal education session. A nonstatistically notable trend also exists toward younger age and increased likelihood of a concomitant distal ulnar fracture in the noneducation trained cohort, which could theoretically facilitate an easier reduction in the noneducation cohort.

The relatively small size of our sample may have been underpowered to detect any true differences that exist. In addition, the curriculum may not have been in place long enough to see an impact clinically. Regarding the education design, the timing was not sufficient for supervised repetition, given the brevity. The session was divided into a lecture and practical session by a week during the resident's pre-existing morning education lecture time. No clinical feedback exists after the session was completed.

In conclusion, a one-time 60-minute education session consisting of a short lecture and practical training session was not sufficient to improve patient outcomes in distal radial fractures. This highlights that although residents may hold their casting ability in high regards, it may not translate to patient outcomes. Educational curricula ought to increase in uniformity and go beyond the “See one, do one, teach one” paradigm. Residency programs directors ought to should make efforts to incorporate education sessions into their curriculums, with adequate time being allotted to afford residents the opportunity to repeatedly practice techniques and providing real-time feedback. Simulation models may be a useful adjunct to provide objective measures for this. Efforts should be focused on longitudinal educational objectives to ensure acquisition and retention of resident skills.

**Appendix:** 

Casting Scoring Sheet *Modified from* ***2015 IPOS Top Gun*** Judge’s Name: ___________________________ Participant’s name/year: ____________________________ **Pre-cast plan-demonstrate proper technique (max 2 points)…………….__________pts** [ ] - demonstrated proper pre-casting/molding plan for dorsally displaced distal radius fracture [ ] – casted wrist flexed and ulnarly deviated **Cast application-demonstrate proper cast application (max 7 points)………..______pts** [ ] – stockinette cut to length with thumb hole [ ] – cast padding applied evenly in 2-3 layers [ ] – finger MCP joints (knuckles of model) are visible under distal edge of cast [ ] – at least 7 cm of thumb is exposed as measured along radial border of thumb [ ] – cast extends proximally within 2 fingers breadth from the antecubital fossa [ ] – fiberglass does not directly contact skin [ ] – proper cast molding applied without indentations and in proper locations **Cast removal-safe use of cast saw (max 4 points)……………………………________pts** [ ] – cast saw is stabilized with finger or thumb [ ] – in-out technique utilized [ ] – cast split to padding circumferentially and spread apart [ ] – no marks identified on the model participant **Total Points……………………………………………………………………_________pts** **FINAL SCORE = (total points/15) x 100……………………………………____________**
